# Comparative Evaluation of YOLOv8 and YOLOv11 for Digital Phenotyping of Edible Mushrooms Under Controlled Cultivation Conditions

**DOI:** 10.3390/jof12040232

**Published:** 2026-03-24

**Authors:** Doo-Ho Choi, Youn-Lee Oh, Minji Oh, Eun-Ji Lee, Sung-I Woo, Minseek Kim, Ji-Hoon Im

**Affiliations:** Mushroom Research Division, National Institute of Horticultural and Herbal Science, RDA, Eumseong 27709, Chungbuk, Republic of Korea; gamchoduho@korea.kr (D.-H.C.); o5ne2@korea.kr (Y.-L.O.); minji1228@korea.kr (M.O.); ejg1105@korea.kr (E.-J.L.); woosungi1013@korea.kr (S.-I.W.); kmstaur@korea.kr (M.K.)

**Keywords:** YOLOv8, YOLOv11, phenotypic analysis, *Pleurotus ostreatus*, *Flammulina velutipes*

## Abstract

Digital phenotyping is increasingly recognized as an essential tool for the quantitative analysis of fungal morphology, particularly in controlled indoor cultivation systems where large numbers of fruiting bodies must be assessed consistently and non-destructively. While YOLOv8-based deep learning approaches have previously been applied in phenotypic analyses of edible mushrooms, the applicability of newer YOLO architectures to fungal phenotyping remains largely unexplored. In this study, we present a controlled-environment digital phenotyping framework for indoor mushroom cultivation and conduct a systematic benchmarking evaluation of YOLOv11 for phenotypic segmentation in comparison with YOLOv8. Using bottle-cultivated *Pleurotus ostreatus* and *Flammulina velutipes* as representative edible basidiomycetes, we performed a controlled comparison of YOLOv8-seg and YOLOv11-seg using identical datasets, preprocessing pipelines, and hyperparameter configurations. The results demonstrate that YOLOv11 achieves segmentation performance comparable to that of YOLOv8 across all evaluated metrics (ΔmAP_50–95_ < 0.01) while substantially reducing computational complexity, including fewer trainable parameters, lower FLOPs, and decreased gradient load. Validation against caliper-based physical measurements revealed moderate, trait-dependent agreement, whereas inter-model consistency between YOLOv8 and YOLOv11 remained consistently high across diverse morphological and segmentation scenarios. These findings suggest that recent developments in object detection architectures can improve computational efficiency without compromising phenotypic measurement fidelity. More broadly, this study highlights the importance of periodically evaluating emerging detection architectures within biological phenotyping pipelines to ensure scalable, sustainable, and high-throughput fungal phenotyping under controlled-environment cultivation systems.

## 1. Introduction

Since ancient times, mushrooms have been valued for their distinctive flavor, economic importance, and medicinal properties [1,2]; to date, more than 15,000 species have been described, approximately 2000 of which are considered edible [3]. Owing to their saprophytic nature, mushrooms can grow on diverse substrates, including soil and food and agricultural residues. Taxonomically, mushrooms are sporulating fungi belonging to the phylum Basidiomycota and the class Agaricomycetes. The structure of each fruiting body is composed of a stipe (stem), pileus (cap), and lamellae (gills), and variation in these external morphological components is directly linked to commercial quality, developmental stage, and cultivation performance [4].

Phenotypes refer to the observable characteristics arising from interactions between genetic background and environmental conditions [5]. In cultivated mushrooms, phenotypic traits such as fruiting body size, shape, and proportional morphology are essential indicators for assessing market quality, breeding outcomes, and cultivation stability. Throughout the history of mushroom domestication, selective breeding has been used to enhance desirable phenotypic traits for edible purposes [6]. The intensification and automation of modern mushroom cultivation systems have increased production efficiency while simultaneously challenging conventional phenotypic evaluation methods; traditional phenotyping approaches remain labor-intensive, time-consuming, and prone to operator-dependent variability, particularly under high-throughput indoor cultivation conditions [7,8]. As a result, there is a growing demand for efficient, objective, and automated phenotyping strategies. In parallel with advances in digital agriculture, artificial intelligence (AI)-based image analysis has emerged as a promising tool for quantitative phenotyping, with increasing relevance for fungal morphology assessment [9,10].

Instance segmentation is a key methodological development, integrating object detection and semantic segmentation to enable pixel-level delineation of biological structures within convolutional neural network (CNN)-based frameworks [11,12]. In plant phenotyping, instance segmentation has been successfully applied to tasks such as disease assessment and yield-related trait extraction [13,14,15]. In fungal research, computer vision approaches based on the You Only Look Once (YOLO) family have been applied to toxic mushroom classification, fruiting body detection, and phenotypic analysis in cultivated systems [16,17,18]. Owing to its favorable balance between inference speed and accuracy, YOLO has become a widely adopted architecture for real-time object detection. Since its initial introduction in 2016 [19], the framework has undergone continuous architectural refinement. Among the Ultralytics YOLO series, several recent variants, including YOLOv5, YOLOv8, and the recently released YOLOv11, provide support for instance segmentation, thereby enabling pixel-level phenotypic analyses of complex biological structures [16].

Beyond accuracy-oriented performance, recent studies on digital phenotyping have emphasized the importance of system-level requirements in AI model selection, particularly under large-scale, automated, and resource-constrained environments. During indoor mushroom cultivation, phenotypic monitoring often requires continuous image acquisition and processing under constrained computational resources [20]. In such contexts, computational efficiency, architectural compactness, and training stability are critical factors that directly affect the scalability and sustainability of phenotyping pipelines. Consequently, architectural suitability and long-term deployability must be accounted for when evaluating phenotyping models, especially for high-throughput fungal morphology analysis. Recent studies on automated cultivation and digital phenotyping further indicate that lightweight deep learning models play a critical role in enabling scalable and context-aware farming systems, especially where real-time perception and continuous operation are required [21].

Although a growing number of studies have explored AI-based phenotypic analysis, systematic investigations focusing on instance segmentation-based phenotyping of cultivated mushrooms remain limited. Existing studies have predominantly relied on YOLOv8 for the detection and phenotypic characterization of fruiting bodies [9,17,22]. However, digital phenotyping systems deployed in controlled cultivation environments require not only segmentation accuracy but also long-term computational efficiency, scalability, and operational stability. Despite recent advances in object detection architectures, including the release of YOLOv11 with improved computational efficiency, the practical suitability of these emerging architectures for high-throughput fungal phenotyping pipelines has not been systematically evaluated in mushroom cultivation systems [23]. To address this gap, we establish a controlled-environment digital phenotyping benchmark using cultivated mushrooms grown in standardized polypropylene bottle systems. Within this framework, YOLOv8 and YOLOv11 are evaluated under identical training conditions to compare segmentation accuracy, agreement with physical ground-truth measurements, and computational efficiency. Through this systematic evaluation, we aim to determine whether recent architectural refinements can enhance the operational efficiency of fungal phenotyping systems while maintaining reliable measurement performance. Such comparative benchmarking provides practical guidance for selecting suitable detection architectures in scalable mushroom phenotyping platforms and supports the sustainable deployment of AI-assisted cultivation monitoring systems under controlled production environments.

## 2. Materials and Methods

### 2.1. Preparation of Mushroom Material and Image Acquisition

In 2024, under controlled-environment conditions, *Pleurotus ostreatus* and *Flammulina velutipes* were cultivated for phenotypic analysis based on established bottle cultivation protocols reported in previous studies [6,7,24]. Both edible mushroom species were grown in 800 mL polypropylene bottles under standardized and controlled conditions. For the cultivation of *P. ostreatus*, a substrate composed of poplar sawdust, beet pulp, and cottonseed meal was prepared in a 50:30:20 (*v*/*v*) ratio. In the case of *F. velutipes*, the cultivation medium consisted of corn cob, rice bran, beet pulp, soybean hull, wheat bran, crushed oyster shell, and waste limestone mixed in a 35:33:10:6:6:6:4 (*v*/*v*) ratio. All media were sterilized at 121 °C for 90 min prior to inoculation. Fruiting bodies were developed at 23 °C and 80–90% relative humidity following conventional controlled bottle cultivation practices. All images presented in this study were acquired from mature fruiting bodies at the commercial harvest stage to ensure consistency in morphological traits and to focus our analysis on phenotypic features directly relevant to market-oriented evaluation.

**Figure 1:**
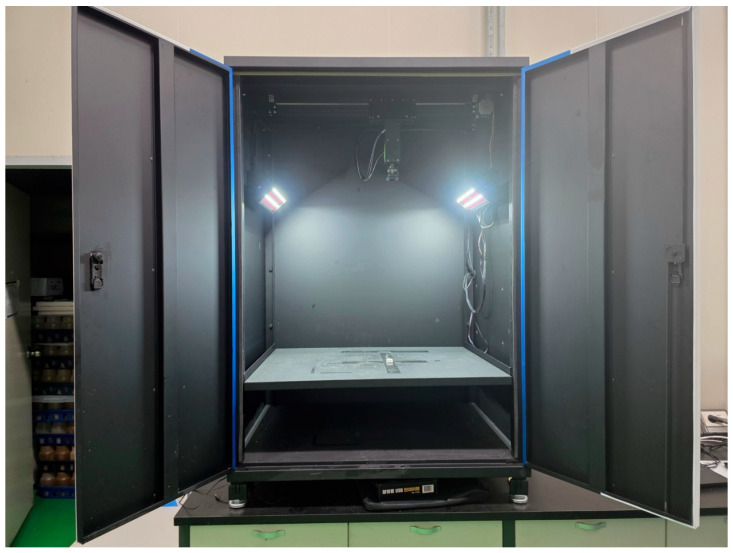
Custom-built imaging chamber for standardized acquisition of mushroom phenotypic data. The chamber was designed to minimize background noise, shadow artifacts, and illumination variability during image capture. Captured images were saved in RGB mode with lossless compression, followed by preprocessing steps, including background normalization, color correction, and resizing to 640 × 640 pixels, for YOLO-based phenotypic analysis.

Side-view images of mushroom fruiting bodies were acquired under strictly controlled illumination and spatial conditions to construct a standardized dataset suitable for deep learning-based digital phenotyping. To minimize background noise, shadow interference, and illumination heterogeneity, a custom-built imaging chamber (PODO Co., Ltd., Korea) was constructed, specifically designed to provide reproducible imaging conditions across all samples. Within the chamber, a high-resolution RGB camera (EOS M50, Canon Inc., Tokyo, Japan) equipped with a Fujinon CF8ZA-1S industrial lens (8 mm focal length, f/1.8) was mounted in a fixed position. Illumination was provided by dual diffused LED panels symmetrically installed at 45° angles relative to the sample plane (Figure 1). During image acquisition, mushroom bottles were placed sequentially at four predefined positions on the chamber floor to account for minor spatial variability while maintaining consistent imaging geometry. These controlled imaging conditions were intentionally adopted to minimize environmental variability and to ensure a fair and reproducible comparison between deep learning models, rather than to replicate all complexities of commercial production environments.

**Figure 2:**
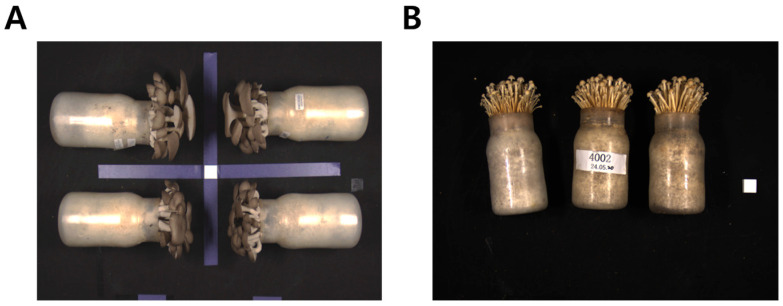
Raw images of *P. ostreatus* (**A**) and *F. velutipes* (**B**). Images illustrate variability in growth stage, individual size, and clustering patterns included in the dataset under controlled conditions in the imaging chamber.

All acquired images were stored in RGB color mode using lossless compression (Figure 2). Image preprocessing was performed using Python-based routines implemented with the OpenCV library (version 4.x). The preprocessing pipeline included background normalization to reduce residual illumination heterogeneity, global color correction based on channel-wise intensity normalization, and resizing of all images to 640 × 640 pixels using bilinear interpolation to comply with YOLO input requirements [25]. No additional data augmentation or manual color manipulation were applied during preprocessing. An identical workflow was applied to all training, validation, and test datasets to ensure reproducibility and fair comparison between deep learning models. Image resizing was performed solely to meet standardized model input requirements and was consistently applied across all datasets, ensuring that relative performance comparisons between models were not biased by preprocessing differences.

### 2.2. Dataset Construction

Following image acquisition, all valid images were organized and preprocessed to construct a reliable dataset for AI model training and evaluation under controlled-environment conditions. The dataset consisted exclusively of mature mushroom fruiting bodies cultivated in bottle-based systems, as mature stages exhibit clearly distinguishable pileus and stipe structures that are essential for instance segmentation-based phenotypic analysis. Although the dataset focused on mature fruiting bodies, substantial morphological variability was preserved, including variations in individual size, stipe length, pileus shape, and degrees of fruiting body overlap, reflecting realistic heterogeneity within controlled indoor cultivation systems.

**Figure 3:**
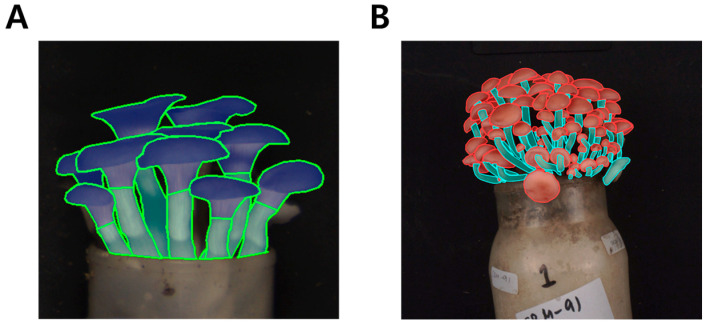
Polygon-based annotation images used for dataset construction: *P. ostreatus* (**A**) and *F. velutipes* (**B**). Pileus and stipe regions were manually annotated using Label Studio to support instance segmentation tasks.

Image annotation was performed on mushroom images (403 side-view images of *P. ostreatus* and 201 of *F. velutipes*) using polygon-based instance segmentation with Label Studio (v1.13, Heartex, San Francisco, CA, USA; https://labelstud.io/, accessed on 10 January 2026) in 2025. The size of our dataset is consistent with previously reported instance segmentation studies in fungal phenotyping, where polygon-based manual annotation substantially limits the feasible scale of annotated images. Two phenotypic categories, pileus and stipe, were manually delineated for each fruiting body (Figure 3). To ensure annotation reliability, all labels were cross-validated by two independent annotators, and discrepancies were resolved through consensus-based review to maintain inter-annotator consistency. The unequal number of images between species reflects inherent differences in cultivation yield and image availability, and was consistently preserved across all subsets to avoid introducing artificial sampling bias.

The annotated dataset was partitioned into training (60%), validation (20%), and test (20%) subsets using stratified random sampling based on predefined morphological categories to ensure balanced representation across subsets [26]. All annotation management and dataset processing steps were conducted using Visual Studio Code (v1.99.3, Microsoft Corp., Redmond, WA, USA; https://code.visualstudio.com/, accessed on 10 January 2026) and integrated within the Ultralytics YOLO framework, ensuring seamless compatibility between the annotation schema and the model input pipeline [16,27]. The final dataset conformed to the COCO-format instance segmentation structure, enabling reproducible and fair comparative analysis across different YOLO architectures. Stratification was performed based on coarse morphological attributes, including relative fruiting body size and the presence of overlapping individuals, rather than species identity, to maintain phenotypic diversity across subsets.

### 2.3. Model Configuration and Training Procedure

Comparative analyses of analytical performance across different YOLO architectures have been reported in various agricultural and phenotyping studies [16,28,29]. In this study, two YOLO-based instance segmentation models, YOLOv8-seg and YOLOv11-seg, were employed for a systematic comparison of mushroom phenotypic segmentation performance. Both models were implemented using the Ultralytics YOLO framework (v8.2.0, Ultralytics Inc., Austin, TX, USA; https://github.com/ultralytics/ultralytics, accessed on 10 January 2026) within the Python 3.12 and PyTorch 2.3.1 environments. All computations were conducted on a Windows 11 workstation equipped with an NVIDIA RTX 4080 GPU (16 GB VRAM, NVIDIA Corp., Santa Clara, CA, USA) and 32 GB RAM, providing sufficient computational capacity for high-resolution instance segmentation.

To ensure controlled and reproducible comparisons between the two architectures, both YOLOv8-seg and YOLOv11-seg were trained under identical hyperparameter settings, following configuration guidelines reported in previous benchmarking studies [8]. Prior to training, all input images were resized to 640 × 640 pixels. The batch size was dynamically adjusted based on available GPU memory, and the learning rate was set to 0.01, the momentum to 0.937, and the weight decay to 0.0005. Stochastic gradient descent (SGD) was used as the optimizer, and training was performed for 120 epochs, including three warm-up epochs to stabilize gradient updates.

Identical hyperparameters were adopted to isolate the effects of architectural differences between YOLOv8 and YOLOv11, rather than to maximize the absolute performance of each model through architecture-specific tuning. Under this controlled setup, observed differences in segmentation accuracy and computational efficiency can be attributed primarily to variations in network design and information flow.

YOLOv8 employs a conventional C2f backbone combined with a spatial pyramid pooling-fast (SPPF) neck, which supports hierarchical multi-scale feature extraction [30]. In contrast, YOLOv11 introduces the C2f-Fusion and RepNCSPELAN modules, which enhance feature reuse and contextual representation while reducing redundant computation [31]. These architectural modifications primarily alter feature aggregation and propagation mechanisms. To maintain fairness in performance evaluations, both YOLOv8 and YOLOv11 were trained using the same datasets, preprocessing pipeline, and hyperparameter configurations, enabling direct comparison of their suitability, for instance in segmentation-based mushroom phenotypic analysis [16].

### 2.4. Evaluation Metrics

Model performance was evaluated under identical training and hyperparameter conditions to ensure direct comparability between architectures. This controlled comparative design was adopted to assess how incremental architectural developments influence phenotypic measurement reliability and computational feasibility within scalable fungal phenotyping systems. Although other instance segmentation frameworks exist, we focused on YOLO-based architectures to maintain methodological consistency within a real-time detection framework relevant to computationally constrained deployment environments. Validation against physical measurements was subsequently performed to examine biological consistency.

#### 2.4.1. Detection and Segmentation Accuracy

Following the training procedure, the YOLO models were quantitatively evaluated using four standard COCO-based metrics: precision (P), recall (R), mAP_50_, and mAP_50–95_. Precision measures the proportion of correctly identified positive detections among all detections, while recall represents the proportion of correctly detected targets among all ground-truth instances. The mean average precision (mAP) was computed using the COCO evaluation protocol, where mAP_50_ corresponds to an intersection-over-union (IoU) threshold of 0.50 and mAP_50–95_ represents the mean value across thresholds from 0.50 to 0.95 at 0.05 increments [32].

$$P=\frac{TP}{TP+FP}\;R=\frac{TP}{TP+FN}\;mAP=\frac{1}{n}\sum_{i=0}^{n}{AP}_{i}\;{AP}_{i}=\int_{0}^{1}p\left(r\right)dr$$

In the equation above, TP, FP, and FN denote true positives, false positives, and false negatives, respectively, and ${AP}_{i}$ is the area under the precision–recall curve for class i. For mask-based segmentation, these metrics were computed on a pixel-wise IoU basis between predicted and ground-truth masks.

To further evaluate segmentation robustness under partial spatial overlap between pileus and stipe regions, performance was additionally assessed on occlusion-affected instances within the test dataset. Occlusion cases were defined as instances in which annotated morphological components exhibited spatial overlap in side-view images. For these instances, the same evaluation metrics described above were recalculated using an identical IoU-based protocol [33].

#### 2.4.2. Evaluation Metrics for Computational Efficiency and Model Complexity

To evaluate the computational efficiency and scalability of the YOLO models, the following model descriptors were quantified using the THOP (PyTorch) library:

- FLOPs (B): total floating-point operations per forward pass, reflecting computational demand;
- Params (M): number of trainable parameters, representing model size and memory usage;
- Gradients (G): number of gradient tensors updated during backpropagation, indicating optimization cost;
- Layers (L): total number of computational blocks.

These metrics were used to quantitatively assess the segmentation accuracy and computational efficiency of the YOLO models. The evaluation process was designed to verify whether architecturally refining YOLOv11 could achieve a lower computational burden and parameter count compared with YOLOv8 while maintaining comparable accuracy across segmentation metrics [34].

#### 2.4.3. Evaluation Framework for Validation Against Physical Measurements

To verify the biological validity of YOLO-derived measurements, the predicted dimensional traits (pileus diameter, pileus thickness, stipe length, and stipe thickness) were compared against physically measured ground-truth data obtained using a digital caliper. All pixel-based outputs were converted into metric units (mm) using a calibration target captured under identical imaging conditions. Based on previous studies, the agreement between predicted and observed measurements was quantified using four statistical indices [35,36]:

1. Pearson’s correlation coefficient (r) to evaluate linear association;
2. Coefficient of determination (R^2^) from simple linear regression to assess explanatory power;
3. Mean absolute error (MAE) to describe the mean magnitude of deviation between predicted and actual values;
4. Mean relative error (MRE) to describe the proportional deviation between predicted and actual values relative to the corresponding physical measurement.

These indices were computed according to the following formulations:

$$r=\;\frac{\sum_{i=1}^{n}\left(y_{i}-\bar{y})(\hat{y_{i}}-\overset{̿}{\hat{y}}\right)}{\sqrt{\sum {\left(y_{i}-\bar{y}\right)}^{2}}\sqrt{\sum {\left(\hat{y_{i}}-\overset{̿}{\hat{y}}\right)}^{2}}},\;R^{2}=1-\;\frac{\sum_{i}{\left(y_{i}-\hat{y_{i}}\right)}^{2}}{\sum_{i}{\left(y_{i}-\bar{y}\right)}^{2}},\;MAE=\;\frac{1}{n}\sum_{i=1}^{n}\left|y_{i}-\hat{y_{i}}\right|,\;MRE=\;\frac{1}{n}\sum_{i=1}^{n}\frac{\left|y_{i}-\hat{y_{i}}\right|}{y_{i}}$$

where $y_{i}$ and $\hat{y_{i}}$ denote the observed (physical measurement) and predicted values, respectively, and *n* represents the number of samples. All statistical analyses were performed using Python 3.12, NumPy 1.26, and SciPy 1.11, and graphical summaries were generated using Matplotlib 3.8 and Pandas 2.2.

#### 2.4.4. Statistical Analysis and Visualization

All performance values, including both accuracy and computational indicators, were analyzed separately for *P. ostreatus* and *F. velutipes*. The mean and standard deviation were calculated for each metric, and independent-sample t-tests were conducted at a 95% confidence level (*p* < 0.05) to evaluate the statistical significance of differences between YOLOv8 and YOLOv11. Correlation analyses were further performed between computational complexity (FLOPs, Params, gradients) and accuracy (mAP_50–95_) to determine the balance between efficiency and precision. FLOPs, Params, and gradient values were visualized as bar graphs, while the learning loss and mAP convergence trends were depicted using epoch-wise line charts to assess stability during training.

## 3. Results

### 3.1. Comparative Performance of YOLOv8 and YOLOv11

Both YOLOv8-seg and YOLOv11-seg successfully detected and segmented pileus and stipe regions across all test images of *Pleurotus ostreatus* and *Flammulina velutipes* under controlled imaging conditions (Table 1). Quantitative evaluation indicated that YOLOv11 achieved a segmentation performance comparable to YOLOv8 across all accuracy metrics, with computational efficiency examined separately in subsequent sections. The dataset included mushrooms with substantial morphological variability, including differences in pileus diameter, stipe length, and fruiting body density, which frequently resulted in overlapping and partially occluded structures in bottle cultivation environments.

To further examine segmentation robustness under the effects of structural occlusion between pileus and stipe, we additionally calculated occlusion-stratified segmentation metrics (seg_mAP_50_ and seg_mAP_50–95_), which are reported in Table 1. These metrics provide additional insight into segmentation stability under partial overlap conditions commonly observed in dense bottle-cultivated mushroom systems.

For *P. ostreatus*, YOLOv8 achieved a precision (P) of 0.80 and recall (R) of 0.79 for bounding-box detection, with a mAP_50_ of 0.85 and mAP_50–95_ of 0.55. Corresponding mask-based segmentation yielded a mAP_50_ of 0.82 and mAP_50–95_ of 0.45. Under occlusion-stratified evaluation, mAP_50–95_ decreased to 0.34 at the aggregated (“all”) level. Under identical training conditions, YOLOv11 produced comparable bounding-box detection results (mAP_50–95_ = 0.55), identical mask-based segmentation performance (mAP_50–95_ = 0.45), and a slightly higher occlusion-stratified mAP_50–95_ of 0.36. Class-wise analysis indicated that stipe regions exhibited a larger reduction under occlusion conditions than pileus regions in both models, suggesting increased sensitivity of elongated structures to boundary ambiguity under partial spatial overlap. Despite this degradation, inter-model differences remained minimal across box-, mask-, and occlusion-aware evaluations.

For *F. velutipes*, both models exhibited relatively lower precision and recall values compared with *P. ostreatus*, likely due to smaller pixel occupancy and increased structural overlap in side-view images. YOLOv8 achieved mAP_50–95_ values of 0.46 for bounding-box detection and 0.23 for mask-based segmentation, with a further decrease to 0.22 under occlusion-stratified conditions. YOLOv11 achieved mAP_50–95_ values of 0.48 (box), 0.24 (mask), and 0.24 under occlusion-aware evaluation. As observed in *P. ostreatus*, stipe regions demonstrated greater performance sensitivity to occlusion than pileus regions. Nevertheless, the relative performance ranking between YOLOv8 and YOLOv11 remained consistent across box-, mask-, and occlusion-aware metrics.

**Figure 4:**
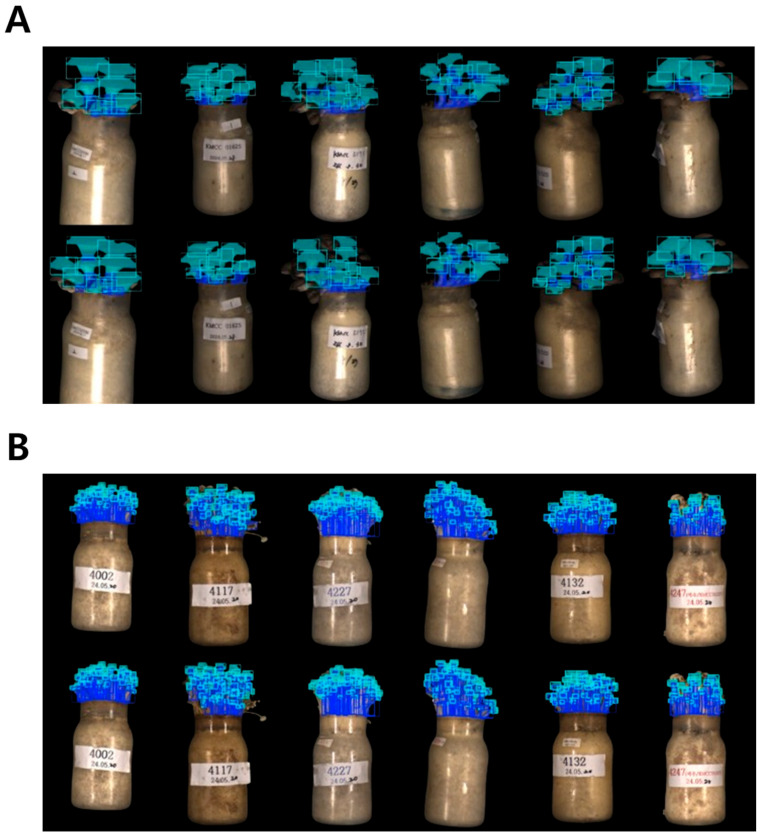
Qualitative comparison of instance segmentation results obtained using YOLOv8 and YOLOv11 for *P. ostreatus* (**A**) and *F. velutipes* (**B**). The top row shows segmentation results generated by YOLOv8, while the bottom row shows the corresponding results generated by YOLOv11. For each species, identical mushroom samples were analyzed by both models to enable direct visual comparison. The images illustrate typical structural characteristics observed in bottle cultivation systems, including overlapping pileus regions, partial occlusion between neighboring fruiting bodies, and elongated stipe structures that introduce boundary ambiguity during segmentation. Despite these structural challenges, both YOLO architectures produced visually comparable segmentation outputs across diverse morphological configurations.

Qualitative inspection of segmentation outputs further demonstrated that both YOLOv8 and YOLOv11 generated consistent mask delineation of pileus and stipe regions across diverse morphological conditions. As shown in Figure 4, both models accurately segmented fruiting bodies with varying sizes and degrees of overlap, producing visually comparable mask boundaries. Although YOLOv11 occasionally produced visually smoother boundary delineation along pileus margins, these differences were qualitative in nature and did not correspond to statistically significant differences in quantitative accuracy metrics.

Overall, our quantitative and qualitative results indicate that YOLOv11 maintains segmentation performance comparable to YOLOv8 at a level comparable to YOLOv8 under controlled-environment conditions, providing a robust phenotypic performance baseline for subsequent evaluation of computational efficiency and model scalability.

### 3.2. Computational Efficiency and Model Complexity

**Figure 5:**
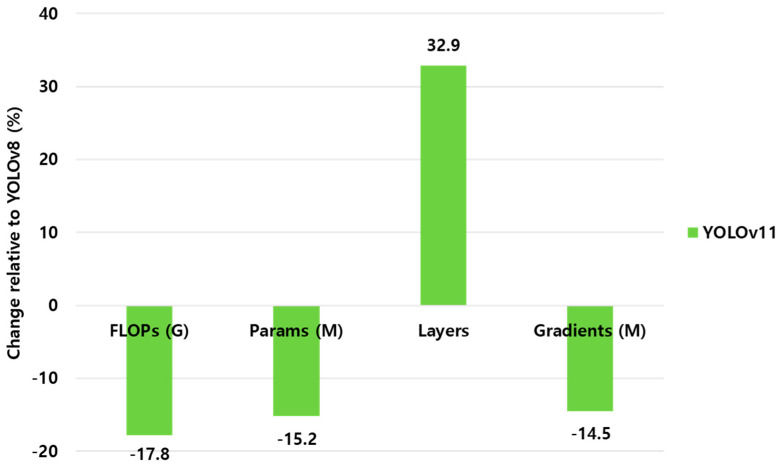
Relative changes in FLOPs, parameters, layers, and gradients of YOLOv11 compared with YOLOv8. YOLOv11 achieved a 17.8% reduction in FLOPs and approximately 15% fewer parameters and gradients, despite an increase in network depth (113 vs. 85 layers).

As illustrated in Figure 5, YOLOv11 demonstrated a clear reduction in computational requirements compared with YOLOv8. The total number of floating-point operations (FLOPs) decreased from 39.9 G to 32.8 G, corresponding to an approximate reduction of 17.8%. Similarly, the number of trainable parameters was reduced from 11.8 M to 10.01 M (15.2% reduction), and the gradient load decreased from 11.79 M to 10.08 M, suggesting reduced optimization overhead during training. These results indicate that YOLOv11 adopts a more compact network configuration with reduced computational and memory demands. Although the total number of network layers increased slightly (from 85 to 113), this reflects architectural restructuring rather than added computational burden, enabled by modular components such as C2f-Fusion and RepNCSPELAN.

**Figure 6:**
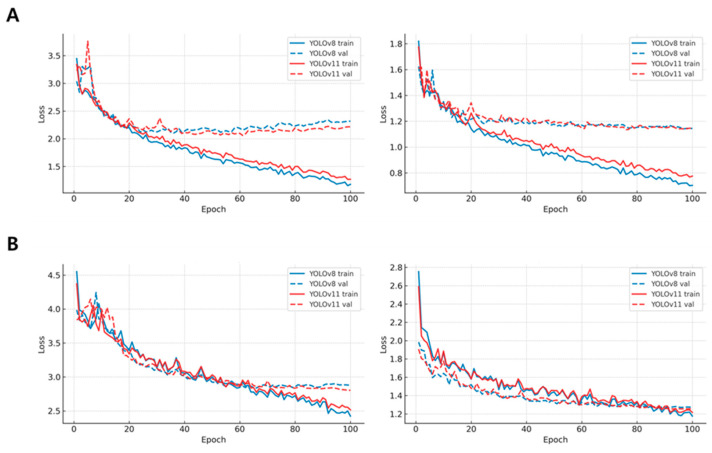
Training and validation loss trends of YOLOv8 and YOLOv11 models for (**A**) *Pleurotus ostreatus* and (**B**) *Flammulina velutipes.* The graphs illustrate the epoch-wise decrease in segmentation (**left**) and bounding-box (**right**) losses for both species, showing stable convergence patterns. YOLOv11 exhibited slightly faster convergence and lower validation loss, indicating improved learning efficiency and robustness during phenotypic segmentation.

Training and validation loss curves are presented in Figure 6. Across both the *P. ostreatus* and *F. velutipes* datasets, YOLOv11 showed slightly faster convergence and marginally lower validation loss compared with YOLOv8, particularly for segmentation loss. Validation loss curves for YOLOv11 reached stable plateaus approximately 15–20% earlier in terms of training epochs than those of YOLOv8. After approximately 60 training epochs, both the box and segmentation losses of YOLOv11 remained consistently 10–18% lower than those observed for YOLOv8. These trends indicate improved training efficiency and convergence stability under identical training conditions, rather than direct gains in segmentation accuracy.

Despite the reduced computational complexity, YOLOv11 maintained a segmentation accuracy comparable to that of YOLOv8 (ΔmAP_50–95_ < 0.01), demonstrating that the observed efficiency gains were not accompanied by a loss of representational capability. Collectively, these results suggest that YOLOv11 provides a favorable balance between segmentation performance and computational efficiency for high-throughput phenotyping under controlled-environment conditions.

### 3.3. Validation Against Physical Measurements

To assess the biological consistency of YOLO-derived phenotypic measurements, model outputs were compared with digital caliper-based physical measurements for four key traits: pileus diameter, pileus thickness, stipe length, and stipe thickness (Table 2 and Table 3). The agreement between predicted and observed values was evaluated using Pearson’s correlation coefficient (r), the coefficient of determination (R^2^), mean absolute error (MAE), and mean relative error (MRE), providing complementary perspectives on association strength, explanatory power, absolute deviation, and proportional error.

For *P. ostreatus* (Table 2), image-derived measurements exhibited trait-dependent levels of agreement with physical measurements. The pileus diameter showed limited correspondence, with YOLOv8 achieving r = 0.20, R^2^ = 0.04, MAE = 5.06 mm, and MRE = 12.43%, while YOLOv11 produced similar values (r = 0.23, R^2^ = 0.05, MAE = 5.02 mm, MRE = 12.40%). The pileus thickness displayed consistently low correlation across both models (r ≈ 0.16–0.17, R^2^ ≈ 0.03), accompanied by moderate relative error (MRE ≈ 20%). In contrast, stipe-related traits demonstrated relatively stronger associations, with r values ranging from 0.39 to 0.43 and the R^2^ exceeding 0.15, while MRE values varied depending on trait scale (7–61%). Despite these differences in absolute agreement with physical measurements, the inter-model consistency between YOLOv8 and YOLOv11 was exceptionally high across all traits (r ≥ 0.93, R^2^ ≥ 0.86, MAE ≤ 1.44 mm, MRE ≤ 5.01%), indicating nearly identical dimensional predictions under identical training conditions.

A comparable trend was observed for *F. velutipes* (Table 3), reflecting its slender morphology and frequent overlap of fruiting bodies. For pileus diameter, moderate agreement with physical measurements was obtained for both YOLOv8 (r = 0.42, R^2^ = 0.18, MAE = 2.01 mm, MRE = 15.61%) and YOLOv11 (r = 0.41, R^2^ = 0.17, MAE = 1.99 mm, MRE = 15.35%). Pileus thickness exhibited limited correspondence across models, accompanied by elevated relative error values (MRE ≈ 74%), reflecting the small absolute measurement scale of this trait. Similarly, stipe thickness showed high proportional error (MRE > 120%), whereas absolute deviations (MAE ≈ 3.7–3.9 mm) remained within a moderate range. Stipe length demonstrated low correlation (r = −0.27), yet relative error remained below 10%. Notably, inter-model reproducibility remained extremely high for all traits (r ≥ 0.94, R^2^ ≥ 0.88, MAE ≤ 1.38 mm, MRE ≤ 3.93%), demonstrating consistent phenotypic interpretation between the two architectures.

Overall, these results indicate that YOLO-derived phenotypic measurements capture consistent morphological trends in a consistent manner across models. Although absolute agreement with physical measurements was moderate and strongly trait-dependent, proportional error analysis (MRE) further revealed that traits with smaller measurement scales exhibited amplified relative deviations. Nevertheless, YOLOv11 reproduced the analytical behavior of YOLOv8 with comparable R^2^, MAE, and MRE values. When considered together with the observed reductions in computational complexity, our findings support the applicability of YOLOv11 as an efficient and non-destructive analytical tool for controlled-environment phenotyping workflows. This comparative framework further provides a practical basis for the periodic re-evaluation of emerging detection models within digital fungal phenotyping pipelines.

## 4. Discussion

In this study, we systematically evaluated two widely adopted YOLO segmentation models, YOLOv8 and YOLOv11, within the context of automated fungal phenotyping [37]. Rather than focusing on algorithmic novelty, the objective was to examine how incremental advances in object detection architectures translate into practical implications for mushroom morphological analysis. Both models successfully identified and segmented pileus and stipe regions under controlled imaging conditions, demonstrating stable performance across diverse morphological configurations of *Pleurotus ostreatus* and *Flammulina velutipes*.

The results indicate that YOLOv11 achieves segmentation accuracy equivalent to YOLOv8 while operating with reduced computational complexity. Although quantitative performance indices such as precision, recall, and mAP_50–95_ showed only marginal differences between models (ΔmAP_50–95_ < 0.01), computational analysis revealed reductions in FLOPs, trainable parameters, and gradient load in YOLOv11 under identical training conditions. These findings suggest that recent architectural refinements primarily influence efficiency and convergence behavior rather than substantially altering final detection accuracy [38,39,40]. The observed reductions in computational load further reflect a comparatively lightweight computational profile of YOLOv11 in practical deployment. From a phenotyping perspective, such efficiency-oriented improvements are particularly relevant in workflows requiring repeated model deployment and large-scale data processing. Our occlusion-stratified evaluation further indicates that both YOLO architectures maintain stable segmentation performance under partial structural overlap between the pileus and stipe, a common challenge in dense mushroom cultivation environments.

This study did not incorporate architectural ablation experiments or module-level modifications, as the primary objective was not to optimize or redesign the underlying segmentation architecture. Instead, the focus was placed on evaluating how incremental architectural evolution within a standardized detection framework translates into phenotypic measurement outcomes under controlled cultivation conditions. Although other state-of-the-art instance segmentation frameworks, such as Mask R-CNN or foundation-model-based approaches, have demonstrated strong performance in general computer vision benchmarks, we specifically prioritized YOLO-based architectures due to their favorable balance between inference speed, deployment simplicity, and computational feasibility in indoor cultivation environments. Therefore, the comparative scope was intentionally constrained to real-time YOLO frameworks to ensure methodological consistency and deployment relevance.

Importantly, the additional occlusion-stratified evaluation conducted in this study provides further insight into segmentation robustness under structurally complex conditions. In bottle-cultivated systems, partial spatial overlap between pileus and stipe regions is unavoidable, particularly in side-view imaging configurations. Representative qualitative examples of these structurally complex segmentation scenarios are illustrated in Figure 4, where overlapping pileus and stipe regions frequently occur within dense bottle-cultivated mushroom clusters. The observed reduction in segmentation accuracy under occlusion conditions confirms that boundary delineation is sensitive to mutual structural interference, especially for elongated stipe regions. Nevertheless, the relative performance consistency between YOLOv8 and YOLOv11 across both standard and occlusion-aware evaluations indicates that architectural refinements in YOLOv11 do not introduce additional susceptibility to overlapping structures. These findings suggest that incremental advancements in general-purpose object detection architectures may yield practical benefits for fungal phenotyping systems without compromising robustness under realistic structural constraints. As deep learning models continue to evolve, computational efficiency, training stability, and scalability may become increasingly critical factors for sustainable deployment in high-throughput biological applications [41,42], underscoring the importance of periodically re-evaluating emerging detection frameworks within phenotyping pipelines.

Validation against physical measurements highlighted the inherent limitations of two-dimensional image-based phenotyping, particularly for thickness-related traits and highly occluded structures. These challenges are particularly pronounced in mushroom phenotyping because dense cultivation systems frequently produce overlapping fruiting bodies and irregular morphological boundaries. Such structural complexity inherently increases segmentation difficulty compared with many plant phenotyping scenarios involving more spatially separated organs. Moderate-to-low Pearson’s r and R^2^ values reflect the geometric ambiguity and scale sensitivity associated with projecting three-dimensional structures onto two-dimensional images [35]. Nevertheless, inter-model consistency between YOLOv8 and YOLOv11 remained exceptionally high across all evaluated traits (r ≥ 0.94, R^2^ ≥ 0.86), indicating that both architectures captured largely comparable phenotypic information despite differences in computational footprint [36].

The inclusion of mean relative error (MRE) provided an additional scale-independent interpretation of measurement deviation. While MAE quantifies absolute differences in millimeters, MRE expresses proportional error relative to trait magnitude, facilitating comparison across traits with distinct measurement ranges. Elevated MRE values observed in thickness-related traits, particularly in *F. velutipes*, likely reflect their small absolute dimensions, where minor deviations produce amplified proportional effects. These observations highlight the importance of interpreting proportional and absolute error metrics jointly when assessing automated phenotyping performance.

Several limitations should be acknowledged. The present models were trained under controlled bottle cultivation conditions, and application to other mushroom species or cultivation environments may require dataset expansion and domain adaptation. Furthermore, despite careful annotation procedures, manual polygon labeling inevitably introduces some subjectivity, particularly in overlapping or poorly defined structural regions. Future integration of multi-view imaging or depth-sensing approaches may help reduce geometric ambiguity and improve quantitative correspondence with physical measurements.

Despite these limitations, the framework presented herein demonstrates the feasibility of integrating continually evolving deep learning models into fungal phenotyping workflows. By systematically benchmarking successive YOLO architectures under biologically realistic cultivation conditions, this study provides practical evidence that incremental advances in general-purpose detection frameworks can directly translate into scalable improvements for automated fungal phenotyping systems. The ability to non-destructively quantify morphological traits within cultivation systems offers practical advantages for monitoring uniformity, yield-related traits, and breeding-relevant characteristics. Crucially, this study emphasizes that phenotyping platforms should not remain static; instead, they should be periodically re-evaluated and optimized in response to ongoing advancements in detection architectures. In this context, YOLOv11, rather than representing a paradigm shift in accuracy, can be seen as an example of how incremental improvements in computational efficiency can enhance the scalability and sustainability of digital mushroom phenotyping systems.

From a methodological perspective, this study contributes to fungal phenotyping research by providing a systematic evaluation framework for integrating emerging object detection architectures into biological measurement pipelines. While we do not propose a new segmentation algorithm, we instead demonstrate how successive deep learning architectures can be rigorously assessed in terms of segmentation robustness, computational efficiency, and agreement with physical measurements within a controlled cultivation system. This type of benchmarking framework is particularly important for digital phenotyping applications, where the practical deployment of evolving AI models must be continuously validated against biological measurement standards.

In summary, the primary contribution of YOLOv11 in this study lies in delivering analytical equivalence to YOLOv8 while reducing computational demand and improving operational efficiency. These findings highlight the importance of continuously aligning fungal phenotyping pipelines with emerging developments in object detection technology to support scalable, high-throughput, and sustainable morphological analysis under controlled-environment conditions.

## 5. Conclusions

In this study, we evaluated the performance of YOLOv8 and YOLOv11 segmentation models within an automated fungal phenotyping framework for *Pleurotus ostreatus* and *Flammulina velutipes* cultivated in bottle-based systems. By enabling the non-destructive extraction of key morphological traits from side-view images, the proposed approach provides a practical alternative to conventional destructive phenotyping methods. Both models demonstrated stable and reliable segmentation of pileus and stipe structures across two morphologically distinct mushroom species.

Quantitative analyses showed that YOLOv11 achieved segmentation accuracy comparable to YOLOv8 (ΔmAP_50–95_ < 0.01) while operating with reduced computational complexity, including lower FLOPs, fewer trainable parameters, and reduced gradient load. High inter-model consistency across all evaluated traits further confirmed that computational refinements in YOLOv11 preserved phenotypic interpretation without compromising analytical reliability.

Comparison with caliper-based physical measurements indicated that image-derived traits captured systematic morphological variation, although correspondence with physical measurements remained trait-dependent due to occlusion effects and geometric constraints inherent to two-dimensional imaging. The incorporation of proportional error analysis further emphasized the importance of scale-aware interpretation when applying automated image-based phenotyping to small morphological traits.

Importantly, these findings suggest that ongoing advancements in object detection architectures can enhance the scalability and sustainability of fungal phenotyping systems even when absolute accuracy gains are modest. In this context, YOLOv11 represents a computationally efficient implementation method that maintains analytical equivalence while offering a comparatively lightweight and scalable solution for high-throughput, non-destructive phenotyping under controlled-environment cultivation conditions. Continuous evaluation and integration of emerging detection models will therefore be essential to ensure that digital phenotyping platforms remain aligned with technological progress.

## Figures and Tables

**Table 1:** Quantitative performance evaluation of YOLOv8 and YOLOv11 for mushroom phenotypic analysis.

Species	Model	Class	Box P	Box R	Box mAP_50_	Box mAP_50–95_	Mask P	Mask R	Mask mAP_50_	Mask mAP_50–95_	Seg P	Seg R	Seg mAP_50_	Seg mAP_50–95_
*Pleurotus* *ostreatus*	YOLOv8	all	0.80	0.79	0.85	0.55	0.81	0.75	0.82	0.45	0.81	0.60	0.72	0.34
		stipe	0.77	0.70	0.77	0.39	0.76	0.64	0.72	0.32	0.72	0.55	0.63	0.25
		pileus	0.84	0.88	0.92	0.72	0.86	0.86	0.92	0.60	0.90	0.64	0.81	0.43
	YOLOv11	all	0.82	0.78	0.84	0.55	0.81	0.75	0.82	0.45	0.82	0.64	0.73	0.36
		stipe	0.79	0.70	0.76	0.38	0.77	0.66	0.73	0.30	0.74	0.60	0.66	0.28
		pileus	0.85	0.85	0.92	0.71	0.85	0.85	0.91	0.59	0.91	0.67	0.80	0.45
*Flammulina* *velutipes*	YOLOv8	all	0.73	0.75	0.77	0.46	0.66	0.65	0.64	0.23	0.66	0.62	0.61	0.22
		stipe	0.70	0.68	0.69	0.38	0.60	0.56	0.53	0.18	0.60	0.51	0.50	0.17
		pileus	0.77	0.82	0.84	0.54	0.71	0.75	0.74	0.27	0.71	0.72	0.71	0.26
	YOLOv11	all	0.72	0.76	0.77	0.48	0.66	0.68	0.66	0.24	0.67	0.65	0.65	0.24
		stipe	0.68	0.67	0.69	0.39	0.60	0.58	0.56	0.19	0.59	0.55	0.54	0.18
		pileus	0.76	0.84	0.85	0.57	0.71	0.77	0.77	0.29	0.75	0.75	0.77	0.31

**Table 2:** Validation of phenotypic trait measurements predicted by YOLO in *Pleurotus ostreatus*.

		Pearson (r)	R-Score (R^2^)	MAE (mm)	MRE (%)
Pileus (diameter)	YOLOv08/ Physical Measurements	0.20	0.04	5.06	12.43
	YOLOv11/ Physical Measurements	0.23	0.05	5.02	12.40
	YOLOv08/ YOLOv11	0.95	0.91	1.16	3.09
Pileus (thickness)	YOLOv08/ Physical Measurements	0.16	0.03	5.68	20.41
	YOLOv11/ Physical Measurements	0.17	0.03	5.54	19.90
	YOLOv08/ YOLOv11	0.94	0.89	0.86	4.07
Stipe (thickness)	YOLOv08/ Physical Measurements	0.43	0.18	4.60	61.46
	YOLOv11/ Physical Measurements	0.39	0.15	4.51	60.42
	YOLOv08/ YOLOv11	0.93	0.86	0.63	5.01
Stipe (length)	YOLOv08/ Physical Measurements	0.42	0.17	9.03	7.82
	YOLOv11/ Physical Measurements	0.43	0.19	8.88	7.70
	YOLOv08/ YOLOv11	0.98	0.96	1.44	1.28

**Table 3:** Validation of phenotypic trait measurements predicted by YOLO in *Flammulina velutipes*.

		Pearson (r)	R-Score (R^2^)	MAE (mm)	MRE (%)
Pileus (diameter)	YOLOv08/ Physical Measurements	0.42	0.18	2.01	15.61
	YOLOv11/ Physical Measurements	0.41	0.17	1.99	15.35
	YOLOv08/ YOLOv11	0.98	0.96	0.22	2.19
Pileus (thickness)	YOLOv08/ Physical Measurements	0.22	0.05	3.23	74.49
	YOLOv11/ Physical Measurements	0.19	0.04	3.23	74.76
	YOLOv08/ YOLOv11	0.98	0.95	0.18	2.25
Stipe (thickness)	YOLOv08/ Physical Measurements	0.39	0.15	3.71	124.93
	YOLOv11/ Physical Measurements	0.39	0.15	3.90	131.22
	YOLOv08/ YOLOv11	0.94	0.88	0.28	3.93
Stipe (length)	YOLOv08/ Physical Measurements	−0.27	0.07	11.19	9.58
	YOLOv11/ Physical Measurements	−0.27	0.07	11.09	9.49
	YOLOv08/ YOLOv11	0.95	0.90	1.38	1.34

## Data Availability

The data supporting the findings of this study are available on Zenodo at https://doi.org/10.5281/zenodo.18372009. Additional data and materials are available from the corresponding authors upon reasonable request.
